# Propofol attenuated liver transplantation-induced acute lung injury via connexin43 gap junction inhibition

**DOI:** 10.1186/s12967-016-0954-1

**Published:** 2016-06-30

**Authors:** Dongdong Yuan, Guangjie Su, Yue Liu, Xinjin Chi, Jiayu Feng, Qianqian Zhu, Jun Cai, Gangjian Luo, Ziqing Hei

**Affiliations:** Department of Anesthesiology, The Third Affiliated Hospital of Sun Yat-sen University, Tianhe Road, Guangzhou, People’s Republic of China

**Keywords:** Liver transplantation, Acute lung injury, Connexin43, Propofol

## Abstract

**Background:**

Postoperative acute lung injury (ALI) is a severe complication after liver transplantation, which influences patient survival rate obviously. However, its mechanisms are unclear and effective therapies are still lacking. The current study focused on effects of propofol on liver transplantation-induced ALI and whether its underlying mechanism was relative with connexin43 (Cx43) alternation. The authors postulated that endotoxin induced enhancement of Cx43 gap junction (GJ) plays a critical role in mediating post liver transplantation ALI and that pretreatment with the anesthetic propofol, known to inhibit gap junction, can confer effective protection.

**Methods:**

Male Sprague–Dawley rats underwent autologous orthotopic liver transplantation (AOLT) in the absence or presence of treatments with the selective Cx43 inhibitor, enanthol (0.1 mg/kg) and propofol (50 mg/kg), a commonly used anesthetic in clinical anesthesia. In vitro study, BEAS-2B cells, a kind of lung epithelial cell line expressing Cx43, exposed to lipopolysaccharide (LPS), which mainly contributed to ALI. Function of Cx43 GJ was regulated by Cx43 specific inhibitors, gap26 (300 μM) or enhancer, retinoic acid (10 μM) and two specific siRNAs.

**Results:**

Compared with the sham group, AOLT results in ALI obviously with plasma endotoxin increase. Cx43 inhibition decreased ALI through inflammatory reaction reduction. In vitro studies, LPS-induced BEAS-2B cells damage was attenuated by Cx43 function inhibition, but amplified by enhancement. Another important finding was propofol reduced Cx43 function and protected against LPS-mediated BEAS-2B cells damage or AOLT-induced ALI, mechanisms of which were also associated with inflammatory reaction decrease.

**Conclusion:**

Cx43 plays a vital role in liver transplantation-induced ALI. Propofol decreased Cx43 function and protected against ALI in vivo and in vitro. This finding provide a new basis for targeted intervention of organ protection in liver transplantation, even in other kinds of operations.

## Background

Liver transplantation is always considered to be the most effective and efficient therapy for patients to cure the end-stage liver disease [1]. However, various kinds of postoperative complications affect patient survival obviously, one of which is just ALI [2]. Causes of ALI is very complex and involve multiple factors, among of which enterogenous endotoxin over-production with its associated inflammatory cytokines explosion is considered to play an important role in this process [3, 4]. During liver transplantation, because inferior vena cava and the portal vein are interrupted, intestinal congestion becomes obvious, which results in intestine motility and barriers destroyed significantly. Both bacterial translocation and enterogenous endotoxin are over-produced, leading to susceptible organs injuries, including lungs [5, 6]. As reported, ALI contributed to mortality of patients suffering from liver transplantation, because patients with ALI prone to develop acute respiratory distress syndrome (ARDS), mortality rate among of which could be as high as 76.5 % [6, 7]. However, mechanisms of this complication are still unclear and effective therapies are lacking. Thus, therapeutic strategies investigation about liver transplantation-induced ALI becomes a new focus in recent years, which is beneficial for patients’ recovery.

As transmembrane proteins, connexins express widely in different kinds of organs and tissues. This big family contains about 21 isoforms and six connexin monomers form a hemichannel on the neighboring cells docking together to form an integral gap junction channel, called gap junction (GJ). Molecule weight of signals less than 1 kDa could be transferred through this special channels, including calcium, cyclic adenosine monophosphate, cyclic guanosine monophosphate, glutathione, etc. Direct signal transfer mediated by GJ always plays an important part in physiological and pathological events [8, 9]. Cx43 (molecular weight is 43 kDa) is one of the most important connexins as far as we know and it is always relative with ALI [10, 11]. As reported, Cx43 and its function alternation could affect the inflammatory factors secretion and intercellular communication, which was involved in the on come and development of pulmonary inflammation and lung edema [12]; others demonstrated that Cx43 mediated spread of Ca^2+^-dependent proinflammatory responses in lung capillaries [13]. Results above provide a clue that Cx43 is important for lung injury, but function of it on liver transplantation-induced ALI is still unknown. That is one of the key point in our investigation.

Propofol, as a commonly used anesthetic in clinical anesthesia, is considered to have positive effects on organ protection, which has been explored for many years, however, its underlying mechanism is still unclear [14–16]. In our previous studies, we had demonstrated that propofol could attenuate liver transplantation-induced acute kidney injury or decrease X-ray induced cellular toxicity through inhibiting GJ composed of Cx32 [9, 17]. Based on these findings, we hypothesized protective effects of propofol were relative with GJ mediation. Thus, in current study, we investigate protective effects of propofol on liver transplantation-induced ALI and whether its underlying mechanism is relative with Cx43 function inhibition. This investigation could confer a new basis for therapy development to combat liver transplantation-mediated ALI.

## Methods

### Animals and treatment

Male Sprague–Dawley rats (200–220 g) were purchased from Sun Yat-Sen University. The study was approved by the Laboratory Animal Care Committee of Sun Yat-Sen University (Guangzhou, Guangdong, China) and animal care followed National Institutes of Health criteria for the care and use of laboratory animals in research. Enanthol (Sigma-Aldrich, St. Louis, MO) was given at 0.1 mg/kg 4 h before AOLT and propofol (Sigma-Aldrich, St. Louis, MO) was given at 50 mg/kg for 3 days intraperitoneally before AOLT. This dosage of propofol has been widely used in in vivo studies in rats and produced beneficial effects. Our previous studies showed that propofol 50 mg/kg, produced a sedative response in rats, as determined by loss of reflex responses to a painful stimulus while remaining sensitive to skin incision [9].

AOLT models of rat were established according to our previous study. Rats were fasted for 8 h with free access to drinking water prior to surgery. Open face guard was used to administer the inhalational ether anesthesia until rats did not have response to a needle stimulus. Subsequent to entering the abdominal cavity, the falciform ligament of the liver was resected and ligated, and the left vena phrenica along the esophagus was severed. The liver was revealed until the supra hepatic vena cava (SVC) was liberated and then, and then placed back into its original position. A bold line was prepared to guide the SVC for blockage easily using vascular clamps at a later stage. After left renal vein upper region was liberated, we dissociated the inferior vena cava (IVC), dissected the first hepatic portal and separated the portal vein (PV) from the convergence of the inferior mesenteric and splenic veins. And then hepatic artery and biliary were both liberated according to their anatomic relationship. Subsequently, we ligated the portal hepatics and used microvascular clamps at the convergence of the inferior mesenteric, splenic veins, hepatic artery, SVC, and IVC. The PV was punctured with a 24-gauge needle for reperfusion. We made one 1-mm incision on the IVC wall as an outflow and injected 4 °C Ringer lactate solution at 2.5 ml/min until the liver color turned yellow and closed the opening of the PV and IVC with 8-0 sutures. PV, SVC, IVC, and hepatic artery were all unclamped. The anhepatic phase lasted for 20 ± 1 min on average [5, 9, 14].

### Assessment of lung damage

Eight hours after AOLT, 2 ml air was injected into the tail vein in order to sacrifice the rats. Lung tissues were removed. The middle lobe of the right lung was weighed on an electronic scale and the inferior lobe was fixed in 10 % buffered formalin, embedded in paraffin for hematoxylin–eosin staining. According to the scores of Franco-Gou et al., the pathology was analyzed blindly. The graded edema of the alveolar mesenchyme, intra-alveolar cell infiltration and alveolar hemorrhage were scored. We measured the wet weight of the superior lobe of the right lung and placed it in an oven for 24 h at 80 °C in order to evaporate. The water content of the lung was calculated as: Water content = (lung wet weight−lung dry weight)/lung wet weight × 100 [18, 19].

### Detection of LPS, tumor necrosis factor-α (TNF-α), interleukin-1β (IL-1β), interleukin-6 (IL-6) and interleukin-8 (IL-8)

LPS, TNF-α, IL-1β, IL-6, and IL-8 are determined by their own assay kits following introductions (Sigma-Aldrich). We standardized the results of cytokines (relative LPS, TNF-α, IL-1β, IL-6, and IL-8) of the experimental sham group in vivo or control group in vitro as 1, and the values of other groups were presented as relative values compared to experimental sham group in vivo or control group in vitro, with the purpose to easily visualize the changes of cytokines (relative LPS, TNF-α, IL-1β, IL-6, and IL-8) resulted from changes of Cx43.

### Western blotting

Western blotting follows the standard procedures as previous studies [20]. Cx43 expression were detected with anti-Cx43 (Sigma-Aldrich; 1:4000) and secondary antibody (Sigma-Aldrich; 1:5000). Anti-β-actin and its corresponding secondary antibody (Sigma-Aldrich) were at 1:4000 [21].

### Immunohistochemical staining

According to the appropriate protocol, immunohistochemical staining was performed in 4 μm paraffinized sections. After being dewaxed and dehydrated, the sections were incubated with 3 % H_2_O_2_, inhibiting endogenous peroxidase activity. The primary antibodies against Cx43 was 1:1000 (Sigma-Aldrich) for 24 h at 4 °C. After incubated with its corresponding secondary antibody (Sigma-Aldrich; 1:4000), samples were observed with a light microscope (EclipseE800, Nikon, Tokyo, Japan) [9].

### Cell culture and treatments

BEAS-2B cells, a kind of lung epithelial cell line was obtained from American Type Culture Collection (Manassas, VA, USA). Cells were grown at 37 °C in an atmosphere of 5 % CO_2_ in air and cultured in DMEM/F-12 supplemented with 10 % fetal bovine serum. BEAS-2B cells were pretreated with connexin channel inhibitors gap26, 300 μM, for 1 h (Sigma-Aldrich, a connexinmimetic peptide) and a Cx43 expression enhancer, retinoic acid (RA) 10 μM, for 24 h (Sigma-Aldrich) before LPS (Sigma-Aldrich, 5 μg/ml, 24 h) treatment. Corresponding solvents of gap26 and RA are DMSO [22, 23].

Propofol was at the concentration of 15 μM for 1 h before being subjected to LPS exposure. This concentration at 15 μM was based on our investigation which showed propofol at 15 μM can profoundly decrease dye coupling and also based on our previous study. This concentration of propofol (15 μM) is in the range of target plasma concentration of propofol 2–4 μg/ml (i.e., 11–22 μM) as used clinically during major surgeries [24, 25].

### ‘‘Parachute’’ dye-coupling assay

‘‘Parachute’’ dye-coupling assay was used to detected functional GJ as described [8, 9].

### Cell counting kit-8 (CCK8) and LDH release assay

Cells growth were determined in 24-well plates with CCK8 assay, which was carried out according to the introduction (Dojindo, Tokyo, Japan). LDH release were detected in 96-well plates with its corresponding kits (Sigma-Aldrich) [26].

### Cx43 knock-down with small interfering RNA (siRNA) transfection

Two specific siRNAs (GCTGGTTACTGGTGACAGA and CCGCAATTACAACAAGCAA, Cx43-siRNA1 and Cx43-siRNA2) targeting Cx43 gene were used to reduced Cx43 expression. A nonspecific Cx43-siRNA (NC as shown in the Fig. 4) was used as controls. Lipofectamine 2000 (Invitrogen, Carlsbad, CA, USA) was used to transfect siRNA according to the manufacturer’s instructions.

### Statistical analysis

Statistical analysis was performed with SPSS 15.0 software (SPSS Inc., Chicago, IL). Multiple comparisons were analyzed with repeated measures one-way ANOVA, which is followed by Tukey post hoc comparisons. A two-tailed P value less than 0.05 was considered statistically significantly different.

## Results

### Inhibition GJ composed of Cx43 alleviated ALI followed AOLT

**Fig. 1:**
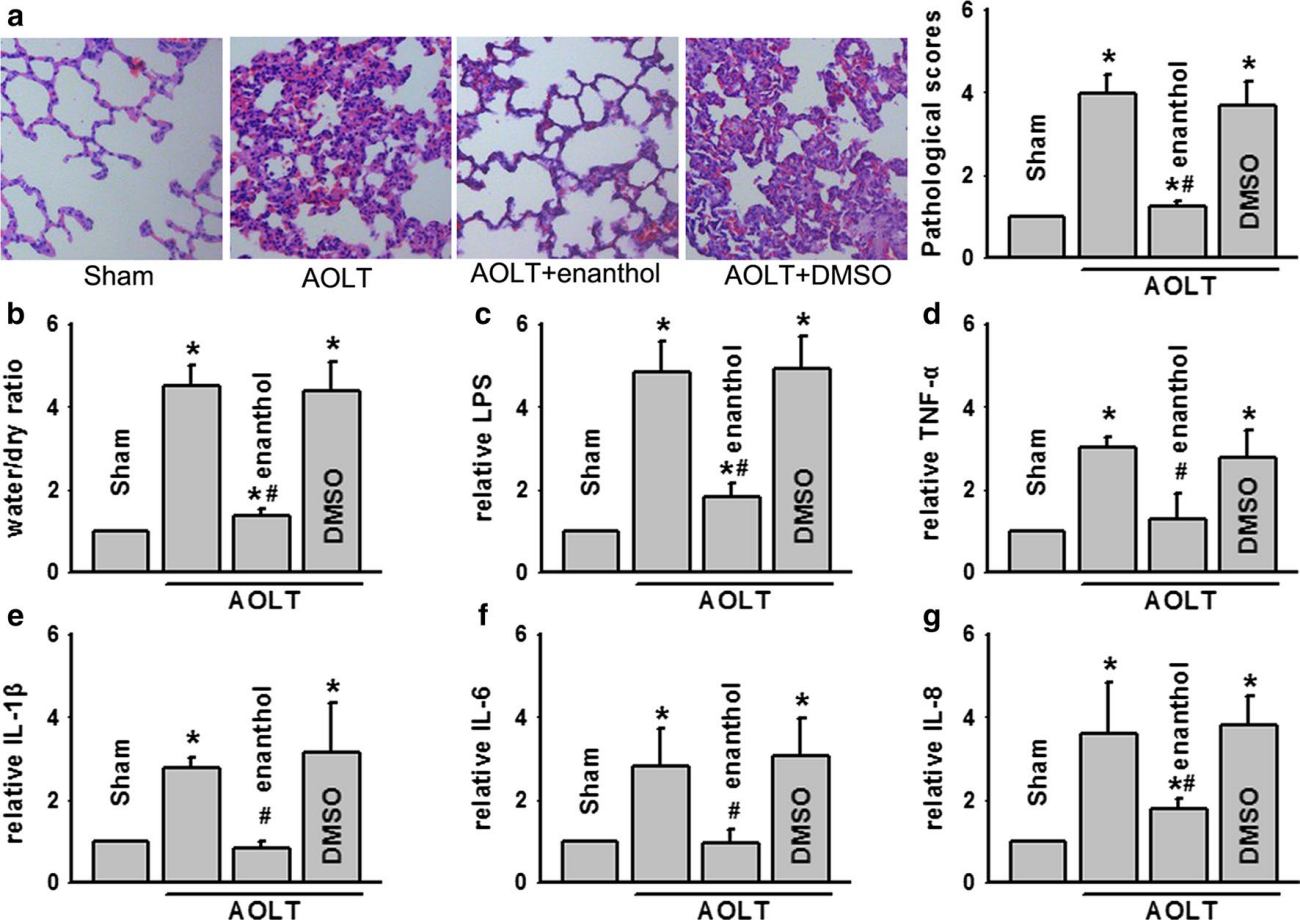
AOLT-induced ALI was attenuated by enanthol application. Rats were exposed to enanthol (0.1 mg/kg) or DMSO for 1 h before AOLT and all samples were obtained 8 h after reperfusion. **a** Pathological damage of lungs (H&E staining; original magnification 200×). **b** Water content of the lung tissues. **c**–**g** Levels of LPS, TNF-α, IL-1β, IL-6 and IL-8 alternation. Vehicle control of enanthol was DMSO, n = 8, *P < 0.05 vs Sham; ^#^P < 0.05 vs AOLT

Rat AOLT model was established to explore effects of liver transplantation on lungs. ALI was most serious at 8 h after AOLT which was the same as our previous study [5]. In AOLT model group, alveolar exudates and inflammatory cell infiltration were obvious; pulmonary interstitium exhibited significant hyperemia and severe hemorrhage. Pathological scores and water content were both higher than that in sham group. Enanthol, a kind of Cx43 inhibitor, alleviated ALI followed AOLT and both pathological scores and water content were improved obviously (Fig. 1a, b).

As reported, intestine motility and barriers were destroyed after liver transplantation, which lead to bacterial translocation and enterogenous endotoxin explosion [5]. Inflammatory cytokines were over produced triggering organ injuries, among of which lungs were the most susceptible organs for endotoxin and inflammatory cytokines [5, 6]. Thus, results in Fig. 1c to g showed that changes of LPS and cytokines were coincident with lung pathological damage after AOLT: LPS, TNF-α, IL-1β, IL-6 and IL-8 were all increased in AOLT model group, which could be reduced by enanthol pretreatment significantly. DMSO, as vehicle control of enanthol had no effects on the parameters.

### Enanthol alleviated lung Cx43 expression followed AOLT

**Fig. 2:**
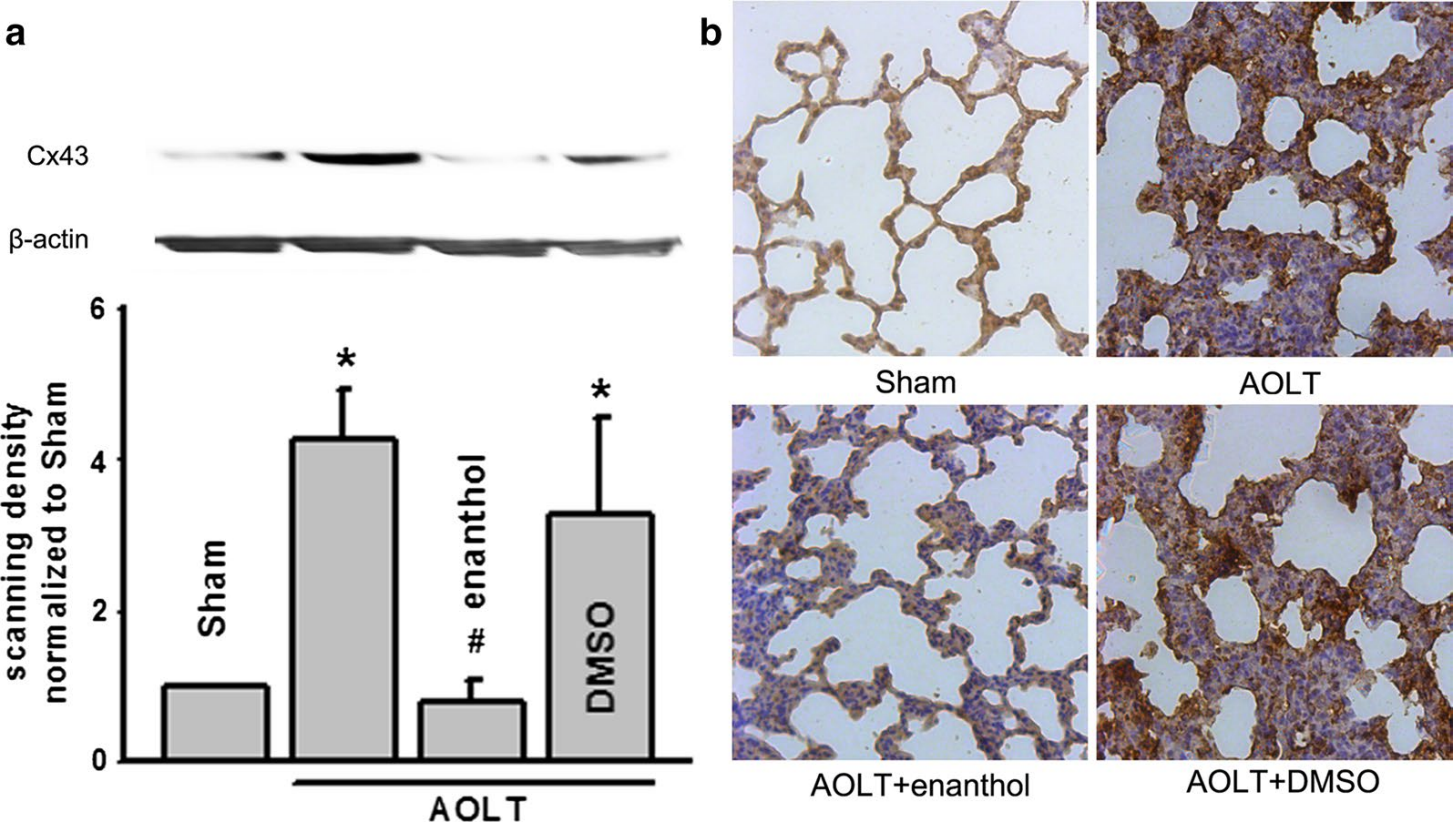
AOLT-induced Cx43 expression increase was attenuated by enanthol application. Rats were exposed to enanthol (0.1 mg/kg) or DMSO for 1 h before AOLT and all samples were obtained 8 h after reperfusion. **a** Cx43 expression of lungs determined with western blotting. **b** Cx43 expression of lungs determined with immunohistochemisty. Vehicle control of enanthol was DMSO, n = 5, *P < 0.05 vs Sham; ^#^P < 0.05 vs AOLT

It has been reported that connexin over-expression always related with organ damage in our previous studies [9, 27]. And results in Fig. 1 also provided a clue that Cx43 might play an important role in AOLT-mediated ALI because of the inhibitor of Cx43, enanthol pretreatment alleviating lung pathological damage and inflammatory cytokines explosion significantly. Thus, we have every reason to suppose that Cx43 expression played an important role in AOLT-mediated ALI. Two different methods, western blotting and immunohistochemisty were carried out to visualize expression and localization of Cx43, both of which showed that Cx43 expression was increased obviously after AOLT, but to be inhibited by the inhibitor of Cx43, enanthol, whereas DMSO, vehicle control of enanthol had no effects on Cx43 expression and localization (Fig. 2). These results were coincident with changes of pathological damage and cytokines explosion in Fig. 1, which supported our hypothesis that Cx43 inhibition attenuated ALI after AOLT via decreasing inflammatory reaction.

### Alternation of Cx43 function influenced LPS-induced BEAS-2B cells damage

**Fig. 3:**
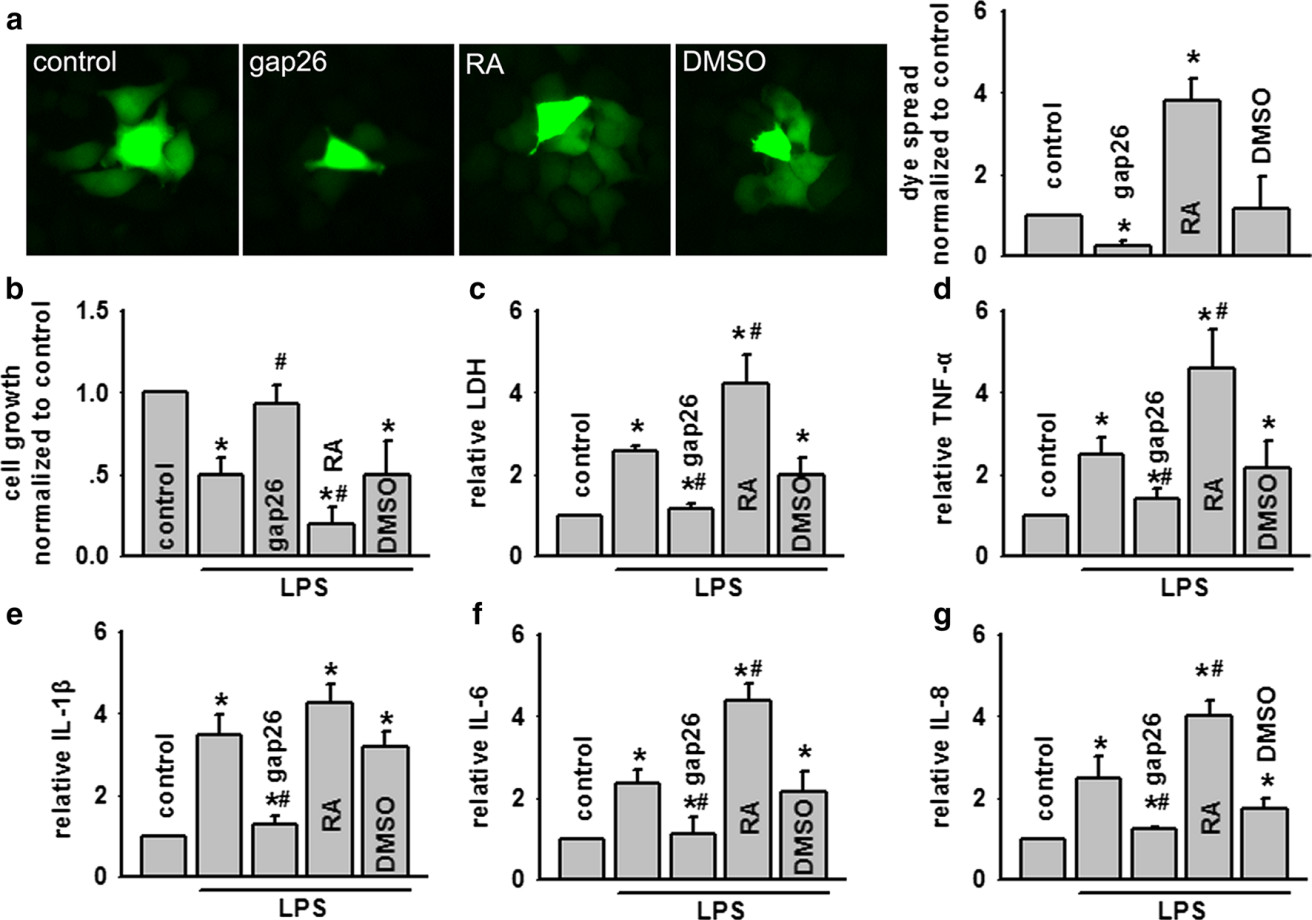
LPS-induced BEAS-2B cells damage was attenuated by enanthol, but amplified by RA. **a** “Parachute” dye-coupling assay was used to determine effects of inhibitor, gap26 (300 μM, for 1 h) and enhancer RA, (10 μM, for 24 h) on GJ composed of Cx43. **b**, **c** Effects of gap26 (300 μM, for 1 h) and RA (10 μM, for 24 h) on cell growth and LDH release exposed to LPS (5 μg/ml, for 24 h), n = 5, *P < 0.05 vs control; ^#^P < 0.05 vs LPS. **d**–**g** Effects of gap26 (300 μM, for 1 h) and RA (10 μM, for 24 h) on TNF-α, IL-1β, IL-6 and IL-8 exposed to LPS (5 μg/ml, for 24 h). Vehicle controls of gap26 and RA were DMSO, n = 5, *P < 0.05 vs control; ^#^P < 0.05 vs LPS

In order to confirm the hypothesis we have mentioned in vivo studies, we further tested it on BEAS-2B cells, a kind of lung epithelial cell line expressing Cx43 [28]. With the facts that endotoxin always played an important role in ALI after AOLT, and the destruction of intestine motility and barriers after liver transplantation lead to endotoxin explosion and inflammatory cytokines over-production, resulting in susceptible organs injuries [29, 30], models of LPS-induced BEAS-2B cells were used to test protective effects of Cx43 inhibition. Dye coupling was reduced by gap26, a specific inhibitor of Cx43, but increased by the enhancer, retinoic acid (RA) (Fig. 3a). At the same time, LPS-induced cell damage was also reduced subsequent to pretreatment with gap26, but exacerbated after RA treatment. Changes in LDH release or other inflammatory cytokines were just opposed to the changes in cell growth. Following pre-incubation with gap26, LDH release, TNF-α, IL-1β, IL-6 and IL-8 were all declined obviously, but increased dramatically with RA pre-treatment. DMSO, vehicle control of gap26 and RA had no effects on the parameters (Fig. 3b–g). These findings suggested that Cx43 GJ played a key role in LPS-induced BEAS-2B cellular damage through mediating inflammatory reaction.

### Cx32 gene knock-down to inhibit Cx32 GJ function prevented LPS-induced BEAS-2B cellular damage

**Fig. 4:**
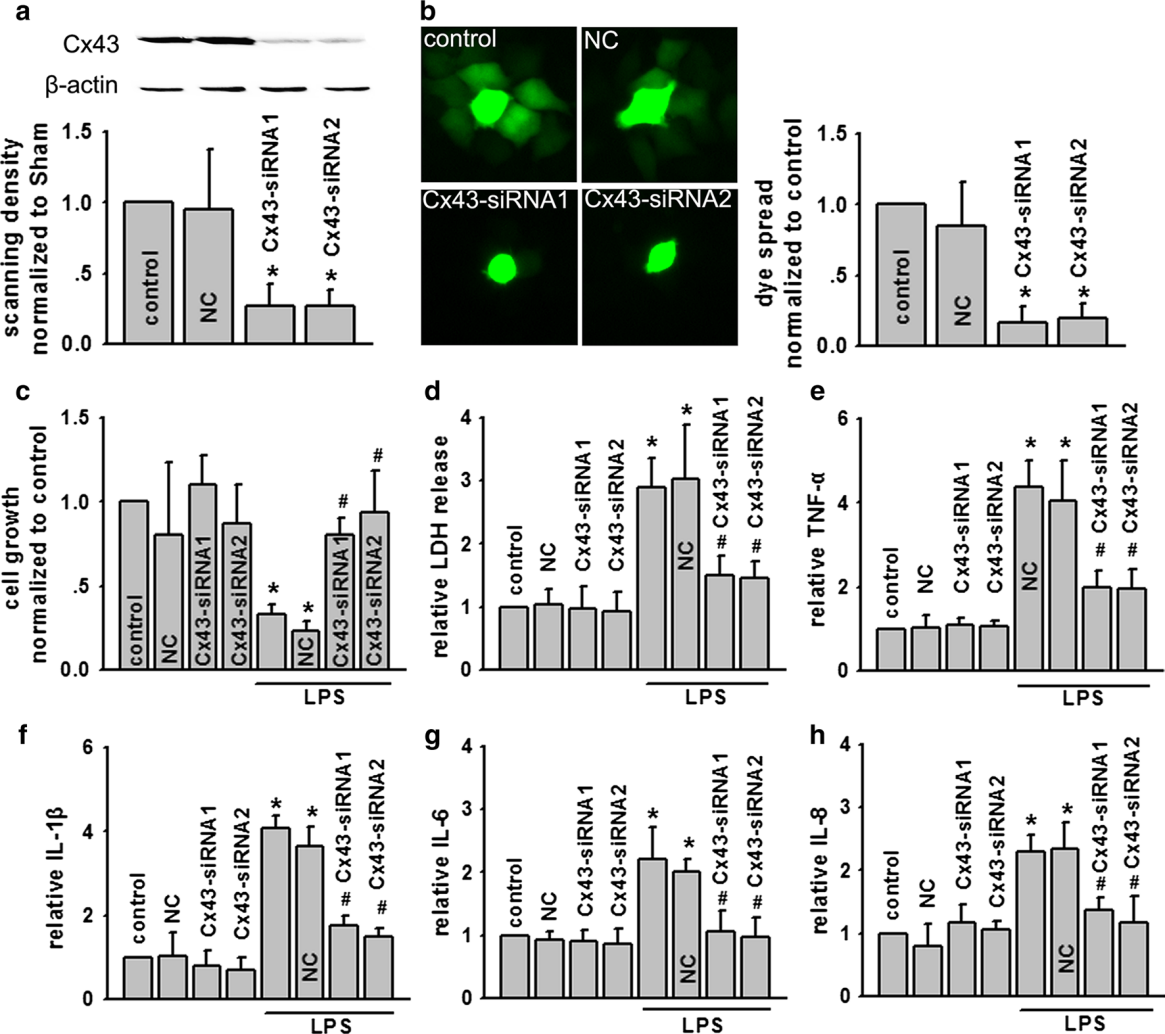
SiRNA targeted Cx43 on BEAS-2B cells attenuated GJ function and LPS-induced cell damage. **a**, **b** Two different specific siRNAs decreased Cx43 expression dye-coupling of BEAS-2B cells. **c**, **d** Effects of siRNAs on cell growth and LDH release exposed to LPS (5 μg/ml, for 24 h), n = 5, *P < 0.05 vs control; ^#^P < 0.05 vs LPS. **e**–**h** Effects of siRNA on TNF-α, IL-1β, IL-6 and IL-8 exposed to LPS (5 μg/ml, for 24 h), n = 5, *P < 0.05 vs control; ^#^P < 0.05 vs LPS

In order to confirm the impact of Cx43 function on LPS-induced BEAS-2B cellular damage, we designed two different siRNAs targeting to Cx43 (Cx43-siRNA1 and Cx43-siRNA2) to specifically knock-down Cx43 expression (Fig. 4a). Blocking Cx43 expression depressed dye coupling of BEAS-2B cells (Fig. 4b). Although Cx43 knock-down per se did not affect cell growth under control condition, it attenuated LPS-induced reduction of cell growth and reduced LDH release significantly (Fig. 4c, d). LPS-induced TNF-α, IL-1β, IL-6 and IL-8 were all inhibited because of siRNAs pretreatment (Fig. 4e, h).

### Propofol attenuated LPS-induced BEAS-2B cells damage via Cx43 inhibition

**Fig. 5:**
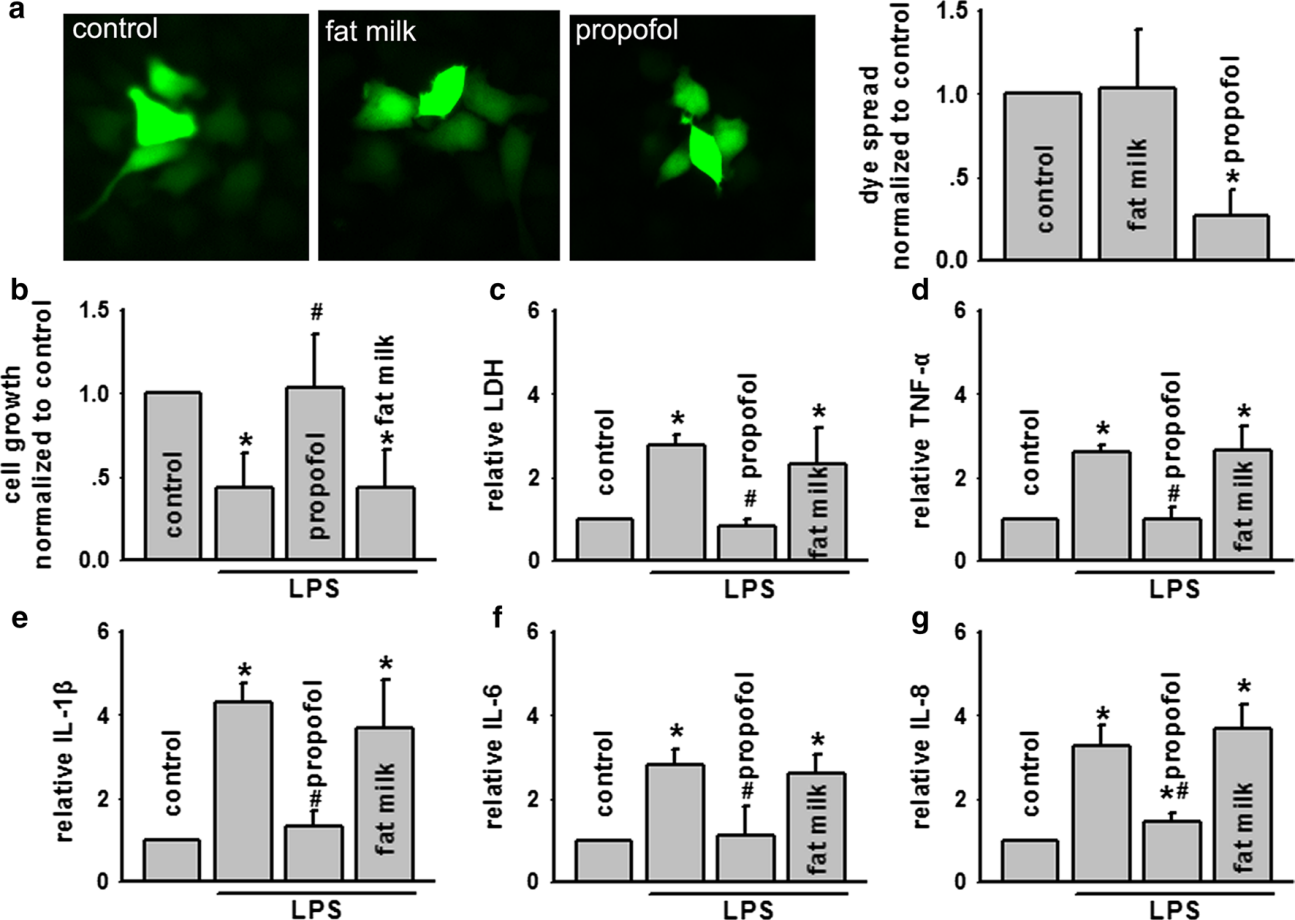
LPS-induced BEAS-2B cells damage was attenuated by propofol. **a** “Parachute” dye-coupling assay was used to determine effects of propofol on GJ composed of Cx43. **b**, **c** Effects of propofol (15 μM, for 1 h) on cell growth and LDH release exposed to LPS (5 μg/ml, for 24 h), n = 5, *P < 0.05 vs control; ^#^P < 0.05 vs LPS. **d**–**g** Effects of propofol (15 μM, for 1 h) on TNF-α, IL-1β, IL-6 and IL-8 exposed to LPS (5 μg/ml, for 24 h). Vehicle control of propofol was fat milk, n = 5, *P < 0.05 vs control;^ #^P < 0.05 vs LPS

Propofol had already been explored for many years and considered to have protective effects on different organs through different mechanisms. In our previous study, we had demonstrated that propofol could attenuate ALI followed AOLT [5], however, whether its mechanism was associated with GJ composed of Cx43 was still unclear. We first examined the effects of propofol on dye coupling of BEAS-2B cells, expressing Cx43. Figure 5a showed that the clinically relevant anesthesia concentration, 15 μM propofol decreased dye coupling of BEAS-2B cells significantly,LPS-induced cell damage was reduced subsequent to pretreatment with propofol, and otherwise LDH release, TNF-α, IL-1β, IL-6 and IL-8 were also declined obviously (Fig. 5b–g). These results showed that propofol attenuated LPS-induced BEAS-2B cell damage through Cx43 GJ inhibition, mechanism of which was relative with inflammatory reaction reduction. Fat milk, vehicle control of propofol had no effects on the parameters (Fig. 5b–g).

### Propofol alleviated ALI followed AOLT via Cx43 inhibition

**Fig. 6:**
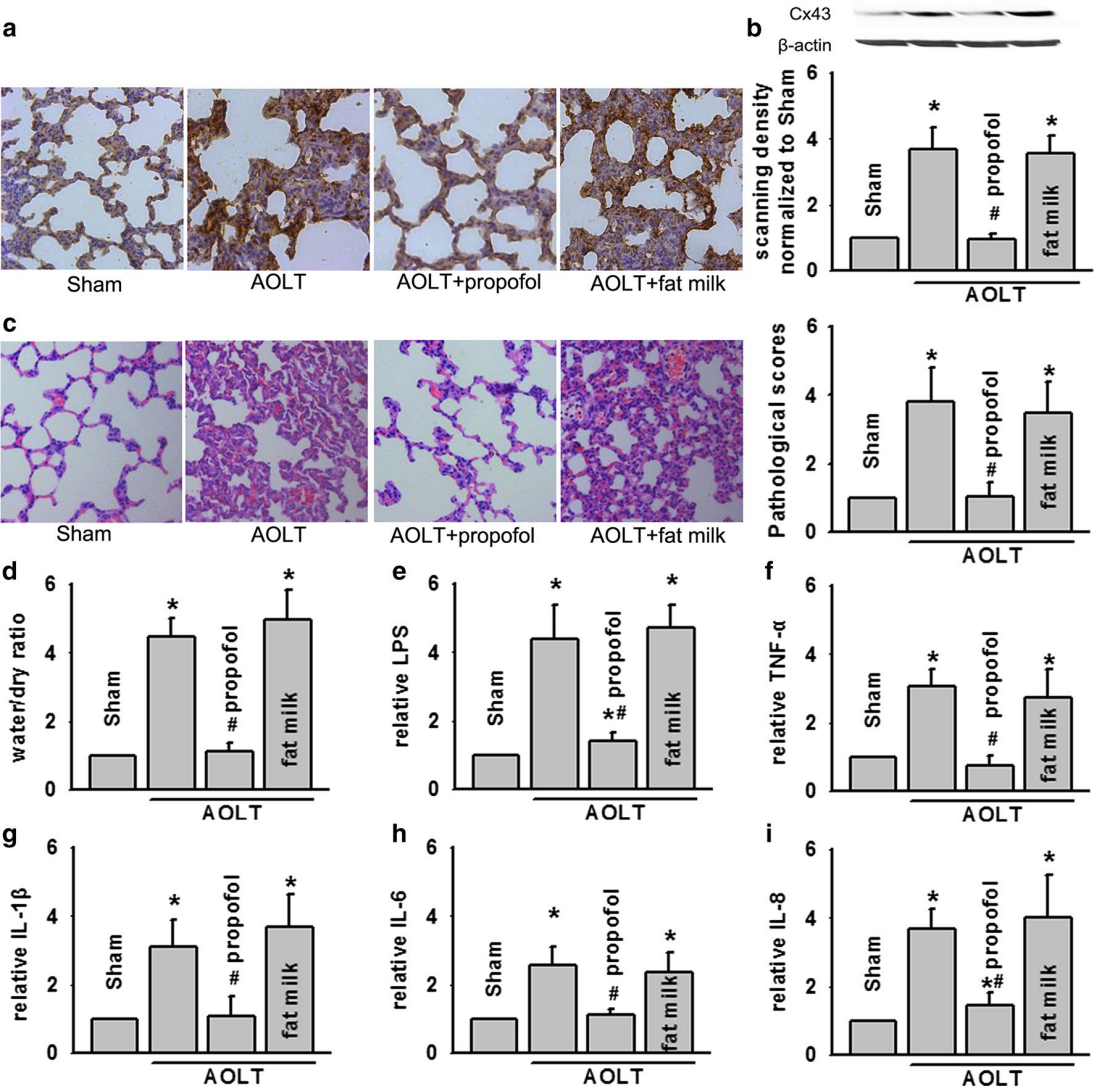
AOLT-induced ALI was attenuated by propofol application. Rats were exposed to propofol (50 mg/kg, intraperitoneally) or DMSO for 3 days before AOLT and all samples were obtained 8 h after reperfusion. **a**, **b** Cx43 expression of lungs determined with immunohistochemisty and western blotting. **c** Pathological damage of lungs (H&E staining; original magnification 200×). **d** Water content of the lung tissues. **e**–**i** Levels of LPS, TNF-α, IL-1β, IL-6 and IL-8 alternation. Vehicle control of propofol was fat milk, n = 6, *P < 0.05 vs Sham; ^#^P < 0.05 vs AOLT

Following our above demonstration in Fig. 5 that propofol attenuated LPS-induced BEAS-2B cells damage via Cx43 inhibition resulting in inflammatory reaction reduction, we wanted to further confirm the protective effects and mechanism of propofol in alleviating ALI followed AOLT in vivo. Both western blotting and immunohistochemisty showed that Cx43 expression was reduced significantly after propofol pretreatment (Fig. 6a, b), which was coincident with lung pathological damage (Fig. 6c, d). Results provided proof that propofol alleviated AOLT-induced ALI through Cx43 inhibition. More importantly, increase of LPS, TNF-α, IL-1β, IL-6 and IL-8 induced by AOLT was attenuated by propofol pretreatment obviously (Fig. 6e, i), whereas fat milk, vehicle control of propofol had no effects on the parameters.

## Discussion

At present, liver transplantation is considered to be the most effective therapy to cure the final-stage liver disease [31]. However, ALI after liver transplantation is severe and influences patient survival significantly [32]. Until now, its underlying mechanism remains unclear. In present study, rat AOLT models were used to explore effects of Cx43 function on liver transplantation-induced ALI, which closely mimicked the most procedure of liver transplantation, such as the SVC, IVC and PV blockade, intestine congestion, cold liver protection, fluid perfusion and liver ischemia reperfusion injury. This model had its own advantages. Compared with allogenic orthotopic liver transplantation, rat AOLT models avoided complex condition of recipients or reject reaction, kept good repeatability and high rat survival rate. From another aspects, rat AOLT model was better than the pure hepatic I/R injury model, because it not only included most procedures of liver transplantation, but also mimicked blood reflux disorder [9]. Thus, this kind of rat model was suitable for us to explore Cx43 GJ effects on ALI after liver transplantation and its potential therapeutic value. During liver transplantation, intestinal congestion was severe because of inferior vena cava and the portal vein interruption, resulting in intestine motility and barriers destroyed significantly. Bacterial translocation and enterogenous endotoxin over-production lead to susceptible organs injuries, such as lungs [33, 34]. It indicated that endotoxin played a central role in liver transplantation-induced ALI. Thus, models of LPS-induced BEAS-2B cells, a kind of lung epithelial cell line expressing Cx43, were used to test protective effects of Cx43 inhibition in vitro.

On rat AOLT models, GJ composed of Cx43 inhibition alleviated ALI, which provided a clue that Cx43 function increase might be a major mechanism of liver transplantation-induced ALI. In vitro study, multiple manipulations regulating Cx43 function were used to further confirm this conclusion. Results showed that function of Cx43 GJ inhibition with specific inhibitor, gap26 or siRNA, alleviated LPS-induced BEAS-2B cells damage; by contrast, Cx43 GJ function increase with a selective enhancer, RA amplified this damage obviously, mechanisms of which were relative with inflammation reaction modulation. We also noticed that propofol, a common anesthetic used in clinic, protected against LPS-induced BEAS-2B cells damage through inhibiting Cx43 channels and that Cx43 inhibition was a major mechanism whereby propofol reduced inflammation reaction under pathological conditions like AOLT-induced ALI.

We had demonstrated that GJ composed of Cx43 played a vital role in AOLT-induced ALI. Others also reported that Cx43 mediated acid-induced increase in microvascular permeability and even regulated spread of Ca^2+^-dependent proinflammatory responses in lung capillaries [13]. GJ composed of connexins mediated direct cell-to-cell transfer of electrical charge or small molecules, which contributed to cell growth, physiology, or response to trauma [35, 36]. Cell signals transferred between neighboring cells always exerted different effects, the most important one of which was just “death signal”, not only directly damaged the neighboring cells, but also activated different signal pathways, resulting in cytotoxicity or apoptosis indirectly [37, 38]. Through this kind of effect, organ damage was amplified significantly, which was called “bystander effect” [39, 40]. Thus, we supposed that “death signal” transfer interruption was just the reason why GJ composed of Cx43 inhibition could protect against liver transplantation-induced ALI effectively. Pity that the intrinsic quality of “death signal” had not been identified. In our previous study, we had explored the possibility of reactive oxygen species (ROS) as “death signal”, but the direct proof was still limited [9]. Although others discussed the possibility of calcium, cells metabolites or molecules, the results were also not satisfied.

In perioperative or preoperative period, proper anesthetics choice was beneficial for patients receiving organ transplantation, because they had protective effects on different organs. We had demonstrated that propofol application, could alleviate rat AOLT-induced ALI, however, the in depth mechanism was still largely unclear [5]; in another investigation, we had clarified that propofol attenuated acute kidney injury via inhibiting GJ composed of Cx32, which indicated GJ inhibition might govern the mechanistic effects of propofol [9]. It had been explored for many years that propofol was always considered to provide protective effects through different mechanisms, such as anti-inflammatory anti-oxidant, or apoptosis inhibition [26, 41, 42]. Incorporating with these facts above, rats were pretreated with propofol to observe its effects on AOLT-induced ALI. Results indicated that propofol alleviated LPS-mediated cell damage through GJ function inhibition in vitro and protected against AOLT-induced ALI also through down-regulating Cx43 GJ function, mechanisms of which were both relative with inflammatory reaction modulation, such as TNF-α, IL-1β, IL-6 and IL-8. Anti-inflammatory effect of propofol had been investigated for many years, however, mechanisms of which were still not understood very well. As far as we known, signals transfer between the neighboring cells were less than 1 kDa, thus, TNF-α, IL-1β, IL-6 and IL-8 (molecular weights were all more than 1 kDa) could not be transferred through GJ directly. Thus, we supposed that “death signal” was over-produced in this process and transferred in the neighboring cells, triggering or amplifying inflammation reaction significantly, which lead to ALI becoming more and more serious. Hence one can see that Cx43 inhibition attenuating inflammation reaction was a potential strategy to protect against liver transplantation-induced ALI. Inhibitory effects of propofol on GJ was obvious in Fig. 5. Thus, we believed that protective effects of propofol on AOLT-induced ALI was relative, or at least partially with function of GJ inhibition. We explained anti-inflammatory effect of propofol from another aspect and provided a valid therapeutic target for liver transplantation-induced organ damage, not just only for lungs.

## Conclusion

Liver transplantation was a serious attack for patients and influenced all of the organs and systems. During liver transplantation, because of inferior vena cava and the portal vein interruption, intestinal congestion resulted in intestine motility and barriers destroyed obviously. Bacterial translocation and enterogenous endotoxin were over-produced and attacked susceptible organs firstly, not just only for lungs [43, 44]. Our present study mainly demonstrated that propofol application protected against AOLT-induced ALI through Cx43 function inhibition, mechanisms of which were relative with inflammatory reaction decrease. These findings might not be universal to all the remote organs, but at least, this conclusion offered a new basis of targeted intervention for organ protection in liver transplantation, even in other kinds of operation.

## Abbreviations

ALI: acute lung injury
Cx43: connexin43
GJ: gap junction
AOLT: autologous orthotopic liver transplantation
LPS: lipopolysaccharide
ARDS: acute respiratory distress syndrome
SVC: supra hepatic vena cava
IVC: inferior vena cava
PV: portal vein
TNF-α: tumor necrosis factor-α
IL-1β: interleukin-1β
IL-6: interleukin-6
IL-8: interleukin-8
RA: retinoic acid
CCK-8: cell counting kit-8
siRNA: small interfering RNA
ROS: reactive oxygen species
